# A Multi-Omic Analysis for Low Bone Mineral Density in Postmenopausal Women Suggests a Relationship between Diet, Metabolites, and Microbiota

**DOI:** 10.3390/microorganisms8111630

**Published:** 2020-10-22

**Authors:** Berenice Palacios-González, Eric G. Ramírez-Salazar, Berenice Rivera-Paredez, Manuel Quiterio, Yvonne N. Flores, Luis Macias-Kauffer, Sofía Moran-Ramos, Edgar Denova-Gutiérrez, Isabel Ibarra-González, Marcela Vela-Amieva, Samuel Canizales-Quinteros, Jorge Salmerón, Rafael Velázquez-Cruz

**Affiliations:** 1 Unidad de Vinculación Científica, Facultad de Medicina, UNAM-INMEGEN, Ciudad de México 04510, Mexico; bpalacios@inmegen.gob.mx; 2 Consejo Nacional de Ciencia y Tecnología (CONACYT)-Laboratorio de Genómica del Metabolismo Óseo, Instituto Nacional de Medicina Genómica (INMEGEN), Ciudad de Mexico 14610, Mexico; eramirez@inmegen.gob.mx; 3 Centro de Investigación en Políticas, Población y Salud de la Facultad de Medicina, UNAM, Ciudad de México 04510, Mexico; bereriveraparedez7@gmail.com (B.R.-P.); jorge.salmec@gmail.com (J.S.); 4 Centro de Investigación en Salud Poblacional, Instituto Nacional de Salud Pública. Cuernavaca, Morelos 62100, Mexico; mquitero@insp.mx; 5 Unidad de Investigación Epidemiológica y en Servicios de Salud, Instituto Mexicano del Seguro Social (IMSS), Cuernavaca, Morelos 62000, Mexico; ynflores@ucla.edu; 6 Department of Health Policy and Management, Center for Cancer Prevention and Control Research and UCLA Kaiser Permanente Center for Health Equity, Fielding School of Public Health and Jonsson Comprehensive Cancer Center, Los Angeles, CA 90095, USA; 7 Unidad de Genómica de Poblaciones Aplicada a la Salud, Facultad de Química, UNAM/Instituto Nacional de Medicina Genómica (INMEGEN), Ciudad de México 14610, Mexico; luisrmacias@gmail.com (L.M.-K.); scanizales@inmegen.gob.mx (S.C.-Q.); 8 Consejo Nacional de Ciencia y Tecnología (CONACYT)- Unidad de Genómica de Poblaciones Aplicada a la Salud, Facultad de Química, UNAM/Instituto Nacional de Medicina Genómica (INMEGEN), Ciudad de México 14610, Mexico; smoran@inmegen.gob.mx; 9 Centro de Investigación en Nutrición y Salud, Instituto Nacional de Salud Pública, Cuernavaca, Morelos 62100, Mexico; edgar.denova@insp.mx; 10 Unidad de Genética de la Nutrición, Instituto de Investigaciones Biomédicas, UNAM, Ciudad de México 04530, Mexico; icig@unam.mx; 11 Laboratorio de Errores Innatos del Metabolismo y Tamiz, Instituto Nacional de Pediatría, Ciudad de México 04530, Mexico; dravelaamieva@yahoo.com; 12 Laboratorio de Genómica del Metabolismo Óseo, Instituto Nacional de Medicina Genómica (INMEGEN), Ciudad de México 14610, Mexico

**Keywords:** inflammation, vitamin D deficiency, lycopene, bone health, intestinal microbiota, postmenopausal women, bone mineral density, estrogen deficiency

## Abstract

The effect of microbiota composition and its health on bone tissue is a novel field for research. However, their associations with bone mineral density (BMD) have not been established in postmenopausal women. The present study investigates the relation of diet, the microbiota composition, and the serum metabolic profile in postmenopausal women with normal-BMD or with low-BMD. Ninety-two Mexican postmenopausal women were classified into normal-BMD (*n* = 34) and low-BMD (*n* = 58). The V4 hypervariable region was sequenced using the Miseq platform. Serum vitamin D was determined by chemiluminescence immunoassay. Serum concentrations of acyl-carnitines and amino acids were determined by electrospray tandem mass spectrometry. Diet was assessed by a food frequency questionnaire. The low-BMD group had fewer observed species, higher abundance of γ-*Proteobacteria*, lower consumption of lycopene, and lower concentrations of leucine, valine, and tyrosine compared with the normal-BMD group. These amino acids correlated positively with the abundance of *Bacteroides*. Lycopene consumption positively correlated with *Oscillospira* and negatively correlated with *Pantoea* genus abundance. Finally, the intestinal microbiota of women with vitamin D deficiency was related to *Erysipelotrichaceae* and *Veillonellaceae* abundance compared to the vitamin D non-deficient group. Associations mediated by the gut microbiota between diet and circulating metabolites with low-BMD were identified.

## 1. Introduction

Osteoporosis (OP) is a skeletal disease characterized by the loss of bone mineral density (BMD) that leads to bone fragility. OP predisposes people to fragility fractures, mainly at the hip, spine, and wrist. The prevalence of OP increases with age and is elevated among postmenopausal women due to estrogen deficiency [1]. In Mexico, the prevalence of osteopenia and OP in 2010 was 32.8% and 8%, respectively, in the population aged ≥40 years [2].

Genetic, hormonal, immune system, nutritional and lifestyle factors play a determining role in bone tissue mineralization. Several mechanisms accelerate bone loss through alteration of osteoblast and osteoclast activity. For instance, bone mass loss mediated by estrogen deficiency is associated with the differentiation of and activity of osteoclasts, due in part to the increase in the production of proinflammatory cytokines (TNF-α, IL-1, IL-6) and the activation of immune cells [3]. Although the mechanism by which these inflammatory processes occur during bone loss is still poorly understood, it has been proposed that intestinal bacteria could lead to the activation of T cells in bone tissue. Among the dietary factors involved in bone health is the consumption of calcium, vitamin D, macronutrients (protein and fat), and micronutrients, such as phosphorus, zinc, magnesium, sodium and potassium, and vitamins (A, E, K, B complex, folic acid, and vitamin C) [4].

The absorption of calcium is determined by several factors: the levels of vitamin D, the intake of Ca, the consumption of phosphates, phytic acid, oxalates, the consumption of excess sodium, and the Ca/phosphorus ratio, among others. Although most of the calcium is absorbed in the small intestine, the colon also has an absorptive capacity. One possible mechanism is the fermentation of dietary fiber by the intestinal microbiota, inducing short-chain fatty acid (SCFA) production, lowering the luminal pH. The low-pH environment increases calcium ions. However, the acidifying medium alone does not ensure calcium transport in the colon. Therefore, attributing the pH reduction entirely to solubilizing the minerals seems like a simplistic approach. Another possible explanation is that SCFAs could directly affect the signaling pathways in the H^+^/Ca^2+^ exchange, regulating the absorption of minerals [5].

Due to its contribution to modulating several host processes, which include energy metabolism, intestinal permeability, intestinal hormone secretion, and the regulation of the immune response, the intestinal microbiota composition has been proposed as an important factor in the development of chronic non-communicable diseases and in several inflammatory alterations, such as Crohn’s disease, obesity, and rheumatoid arthritis [6]. Thus, intestinal dysbiosis impacts the serum metabolome and contributes to the development of insulin resistance (IR); for example, *Prevotella copri* and *Bacteroides vulgatus* were associated with increased concentrations of branched-chain amino acids (BCAAs) and with the presence of IR [7]. Furthermore, intestinal bacteria are involved in the metabolism of several amino acids, which have been implicated in diabetes and cardiovascular diseases [8]. Otherwise, some of the metabolites produced by intestinal bacteria also participate in modulating the immune response. 

Some studies have recently shown a tight relationship between bone metabolism and intestinal microbiota, hence their potential effect on the risk of developing osteoporosis [9,10,11]. However, studies are delimited to populations of Chinese and Europeans. The variability in the microbial profiles and its impact over the health and pathological status in other parts of the world are still unknown. Mainly, the Latin American population is underrepresented [12]. Hence, care must be taken in extrapolating the findings to other ethnic groups. 

Accordingly, the study of the microbiota composition and the health of the bone tissue is a novel field for research, representing a potential new biomarker for diagnosis and prognosis of metabolic diseases in the early stages, as well as contributing to improvements in the treatment [13]. This study aimed to analyze the interactions between diet, intestinal microbiota composition, and serum metabolites in Mexican postmenopausal women with low BMD.

## 2. Materials and Methods 

### 2.1. Study Subjects

Ninety-two unrelated postmenopausal women were selected from “The Health Workers Cohort Study (HWCS),” from the Mexican Social Security Institute (IMSS), located in Cuernavaca, Morelos [14]. Data for this study was collected by the third assessment (January–December 2018) of the HWCS population performed from 2016 to 2018. All enrolled participants answered a questionnaire for the sociodemographic characteristics (birth date, education level, medical history, medical history of relatives, medication use, diet, physical activity, smoking status, alcohol consumption) [15].

All women selected for the analysis accomplished the inclusion criteria (≥45 years of age, postmenopausal status (12 consecutive months without menstruation), and available bone mineral density measurements). Women with metabolic diseases (chronic liver diseases, rheumatoid arthritis, collagen diseases) and those under medication therapy (corticosteroids, anticonvulsants, bisphosphonates, or hormone replacement therapy) were excluded from the analysis [16]. The protocol was approved by IMSS (No. 12CEI 09 006 14, 17 may 2016), and the National Institute of Genomic Medicine (314-07/2017/I, 05 August 2018), following the Declaration of Helsinki (13/LO/0078). All participants provided written informed consent. For an adequate representation of the age variable, we included comparison groups of middle-aged women (ages 45–59 years), middle-old women (60–74 years), and old women (>74 years) [17].

### 2.2. Anthropometric and Clinical Parameters

All parameters measured were performed following standardized procedures. Femoral neck and lumbar spine BMD (g/cm^2^) were calculated using the software in a Lunar DPX NT dual-energy X-ray absorptiometry (DXA) instrument (Lunar Radiation Corp., Madison, WI, USA). All subjects in the analysis were classified into two groups according to the hip T-score measurement. Normal-BMD group included the participants with T-scores from −1.0 to +1, and the low-BMD group included participants with T-scores from −1.1 to −4. Serum vitamin D was determined by the chemiluminescence immunoassay (CLIA) in a LIAISON Analyzer (DiaSorin S.p.A, Saluggia, Italy). Based on the serum vitamin D levels, two groups were performed. The vitamin D deficiency (VDD) group was defined as serum 25 hydroxyvitamin D (25(OH)D) levels <20 ng/mL, and the vitamin D non-deficient (VDND) group as serum 25(OH)D ≥20 ng/mL, as previously reported [15]. To determine body mass index (BMI) (kg/m^2^), the following anthropometric measurements were obtained: weight and height following standardized procedures applied by trained personnel.

### 2.3. Dietary Assessment 

Diet evaluation was performed by a 116-item semi-quantitative food frequency questionnaire (FFQ) derived from a study reported previously for the Mexican population [18,19]. The questionnaire specified a commonly used unit or portion size of food, and frequency of consumption during the past year. The nutrients intake was calculated by multiplying the consumption frequency of each unit of food by the nutrient content according to food-composition tables from the National Institute of Public Health (INSP, by its acronym in Spanish) [19].

### 2.4. Stool Sampling and DNA Extraction 

Each woman was instructed to collect a fecal sample in a sterile container and were then transported in ice-filled coolers. The samples were processed as previously described [20]. Briefly, 200 mg of feces were taken and then stored at −80 °C. Later, bacterial DNA was obtained by a QIAamp DNA stool kit (QIAGEN, Hilden, Germany) following the instructions of the manufacturer. A NanoDrop V3.8.1 spectrophotometer was employed to determine the DNA concentration.

### 2.5. Sequencing of 16S rRNA and Data Analysis 

The specific primers 515F and 806R were used for the sequencing of the V4 hypervariable region, as suggested by the Earth Microbiome Project [21] and as previously described [22]. DNA libraries was sequenced at the Sequencing Unit in the National Institute of Genomic Medicine (INMEGEN) by an Illumina Miseq 2 × 250 platform (Illumina, San Diego, CA, USA) [23]. 

Processing of the Illumina fastq reads was performed using the QIIME 1.8.(16). The UCHIME algorithm was used for detection and removal of chimeric sequences. The Greengenes database was used as the reference 16S database. Taxonomy was assigned by the Ribosomal Database Project (RDP) classifier using a threshold of 80% [24]. The taxonomic composition of the gut microbiota was analyzed by METAGENassist. 

### 2.6. Metabolomics Analysis

Concentrations of serum acyl-carnitines, free carnitine, and amino acids were measured using the approach of targeted metabolomics by electrospray tandem mass spectrometry (quattro Micro API tandem MS, Waters Inc., Milford, MA, USA). Metabolite levels in serum were analyzed using the commercial kit (NeoBase Non-derivatized MS/MS Kit, Perkin Elmer, Waltham, MA, USA), as previously described [25]. Briefly, 20 µL of serum were poured onto filter paper cards (Whatman 903, Dassel, Germany) and dried at room temperature. The spot was cut into 2-mm circles and placed in a 96-well plate. The extraction solution was added to the plate and incubated for 30 min at 30 °C at 650× *g*. Finally, 10 µL of each sample were injected into the flow at 4-min intervals. The Micromass Quattro equipment (Waters Inc., Milford, MA, USA) was used coupled to an ESI source in positive mode. Nitrogen gas was used for desolvation and nebulization, and argon as the collision gas.

### 2.7. Statistical Analysis 

Student’s *t*-test was used to evaluate the parametric variables. For the non-parametric variables, the Kruskal–Wallis test was used to determine the significant variables among the groups. The results were expressed as mean and median. *p*-value < 0.05 were considered as statistically significant. Plots were generated using the ggplot2 package. Differential metabolite levels were evaluated by PLS-DA between groups. Student’s t-tests was performed for the differences between BMD high and BMD low. The Galaxy interface (https://huttenhower.sph.harvard.edu/galaxy) was used to perform a linear discriminant analysis effect size (LEfSe) method for assessment of microbial communities’ differences (LDA < 2). The web-based tool Microbiome Analyst (http://www.microbiomeanalyst.ca) was used to performed heat trees. The heat trees analysis uses the hierarchical structure of taxonomic classifications to quantitatively (using the median abundance) and statistically (using the non-parametric Wilcoxon Rank Sum test) Benjamin Hochberg (FD) corrected depict taxonomic differences between microbial communities.

## 3. Results

### 3.1. Characteristics of the Study Population

Significant differences among the groups in age, femoral neck (FN-) BMD, lumbar spine (LS-) BMD, and total cholesterol (all *p* < 0.05) were found. No significant differences were achieved between both groups in variables, such as BMI, nutritional status, waist circumference, body fat proportion, physical activity, smoking, glucose, diabetes status, vitamin D status, vitamin D intake, calcium intake, and dietary measurements. However, hemicellulose and dietary fiber showed a statistically significant difference. Additional characteristics of the postmenopausal women included in this study are shown in Table 1.

### 3.2. Microbiota Characterization

**Figure 1:**
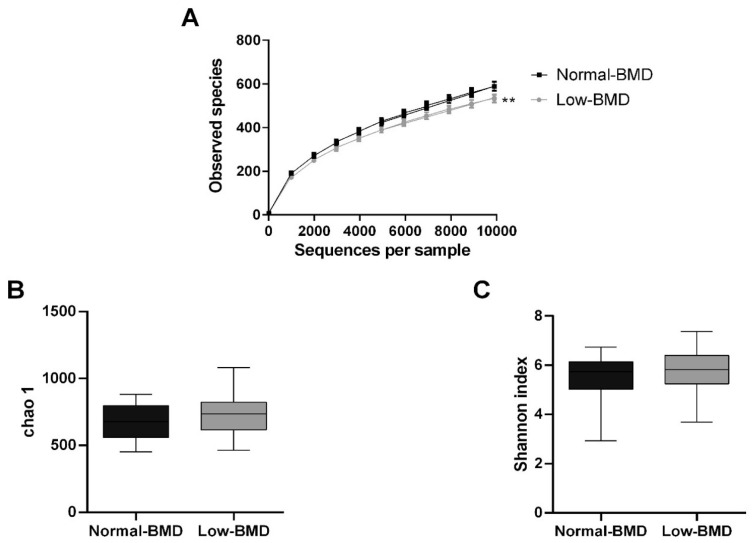
Alpha microbiota diversity in normal-BMD and low-BMD postmenopausal women. (**A**) Alpha rarefaction curves representing the observed number of species in the two study groups. The *y*-axis indicates less observed species in the low-BMD group than the normal-BMD group. No significant differences were found between the groups in richness (Chao index) (**B**) or diversity (Shannon index) (**C**). ** *p* < 0.01 compared to the normal-BMD group.

A total of 4,996,713 high-quality sequences among the 92 fecal samples were generated, with an average of 53,728 sequences per sample. Chao1, Shannon index, and observed species were used for the analysis of alpha diversity. Compared with normal-BMD, the low-BMD group had less observed species (*p* = 0.0002) (Figure 1A). Concerning Chao and Shannon’s diversity indices, no significant differences between the two groups were found (*p* = 0.13 and *p* = 0.18, respectively) (Figure 1B,C).

**Figure 2:**
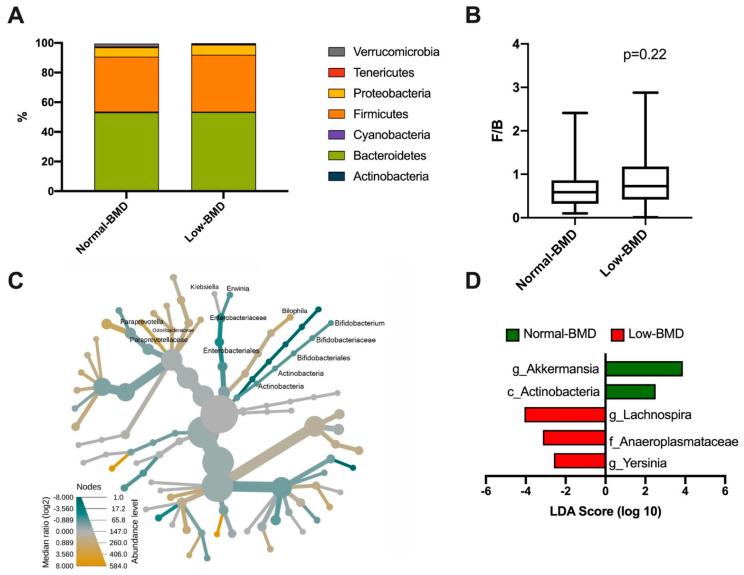
Bacterial taxonomy in normal-BMD and low-BMD postmenopausal women. A total of seven phyla dominated the microbiota composition in both groups, the predominant phyla were *Bacteroidetes*, colored green, followed by *Firmicutes*, colored orange (**A**). About the *Firmicutes*/*Bacteroidetes* (F/B) ratio, no statistical difference between the groups was found (**B**) (*p* = 0.22). (**C**) Heat tree for pair-wise comparison. The color of each taxon indicates the log-2 ratio of proportions observed in each condition. Only those taxa that were statistically significant using the Wilcox rank sum test followed by a Benjamin–Hochberg correction (FDR) for multiple comparisons are shown colored. γ-*Proteobacteria* colored green, specifically the *Klebsiella* and *Erwinia* genus, were enriched in the low-BMD group; meanwhile, the *Bilophila* and *Bacteroidales* group colored yellow were enriched in the normal-BMD group. (**D**) Bacterial taxa differentially represented between groups identified by the linear discriminant analysis (LDA) effect size. Only taxa with an alpha value of 0.05 and with an LDA score of at least 2 are shown. *Lachnospira*, *Anaeroplasmataceae*, and *Yersinia* were enriched in the low-BMD group, while *Akkermansia* and *Actinobacteria* were overrepresented in the normal-BMD group.

A total of seven phyla dominated the microbiota composition. The predominant phyla were *Bacteroidetes*, with a mean abundance of 53% across the dataset, followed by *Firmicutes* with 40%. Other phyla, such as *Proteobacteria*, *Verrucomicrobia*, *Tenericutes*, and *Cyanobacteria*, accounted for 6%, while *Actinobacteria* was less abundant (1%) (Figure 2A). Regarding the *Firmicutes*/*Bacteroidetes* ratio (F/B), although the low-BMD group showed a higher F/B ratio than the normal-BMD group, this was not statistically significant (*p* = 0.22) (Figure 2B). The intestinal microbiota of postmenopausal women in the low-BMD group had significantly higher proportions of γ-*Proteobacteria*, specifically in the *Klebsiella* and *Erwinia* genus, and significantly lower proportions of *Bilophila*, and *Bacteroidales* especially in the family *Paraprevotellaceae*; genus *Paraprevotella* and family *Odoribacteraceae* compared to the normal-BMD group (Figure 2C). To analyze the enrichment of any taxa between two groups, an analysis using the LEfSe algorithm was performed. Enrichment of *Lachnospira* (genus), *Anaeroplasmataceae* (family), and *Yersinia* (genus) was found in the low-BMD group (LDA score ≥ 4, LDA score ≥ 3, and LDA score ≥ 2, respectively). In contrast, an over-representation of *Akkermansia* (genus) and *Actinobacteria* (class) were found in the normal-BMD group (LDA score ≥ 4 and LDA score ≥ 2, respectively) (Figure 2D).

### 3.3. Effects of Age-Related BMD on Bacterial Community Composition

**Figure 3:**
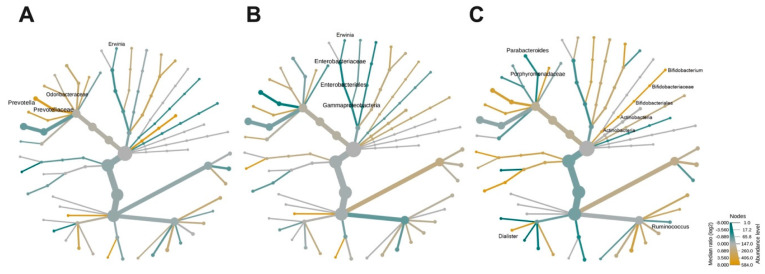
Effects of age-related BMD on bacterial community composition. Heat tree for pair-wise comparison, divided by BMD-age, (**A**) middle-age women (ages 45–59 years), (**B**) middle-old women (60–74 years), and (**C**) old women (>74 years). The color of each taxon indicates the log-2 ratio of proportions observed in each condition. Only those taxa that were statistically significant using the Wilcox rank sum test followed by a Benjamin–Hochberg correction (FDR) for multiple comparisons are shown as colored. Taxa colored green are enriched in the low-BMD group and those colored yellow are enriched in the normal-BMD group.

Age was a differential component between the study groups; hence, the age-associated effect over the composition of the microbiota was assessed. For this, the women were divided into groups of middle-age (ages 45–59 years), middle-old (60–74 years), and old (>74 years) as well as normal-BMD and low-BMD. *Prevotellaceae* and *Prevotella* were decreased in middle-age women with low-BMD compared to middle-age women with normal-BMD (Figure 3A). The γ-*Proteobacteria* clade was enriched in middle-old women with low-BMD when compared to women of the same age with normal-BMD (Figure 3B). Similarly, *Bifidobacterium* was decreased; meanwhile, *Parabacteroides*, *Ruminococcus*, and *Bilophila* were enriched in middle-old women with low-BMD relative to middle-old women with normal-BMD (Figure 3C).

### 3.4. Contribution of Dietary Components to the Composition of Gut Microbiota

**Figure 4:**
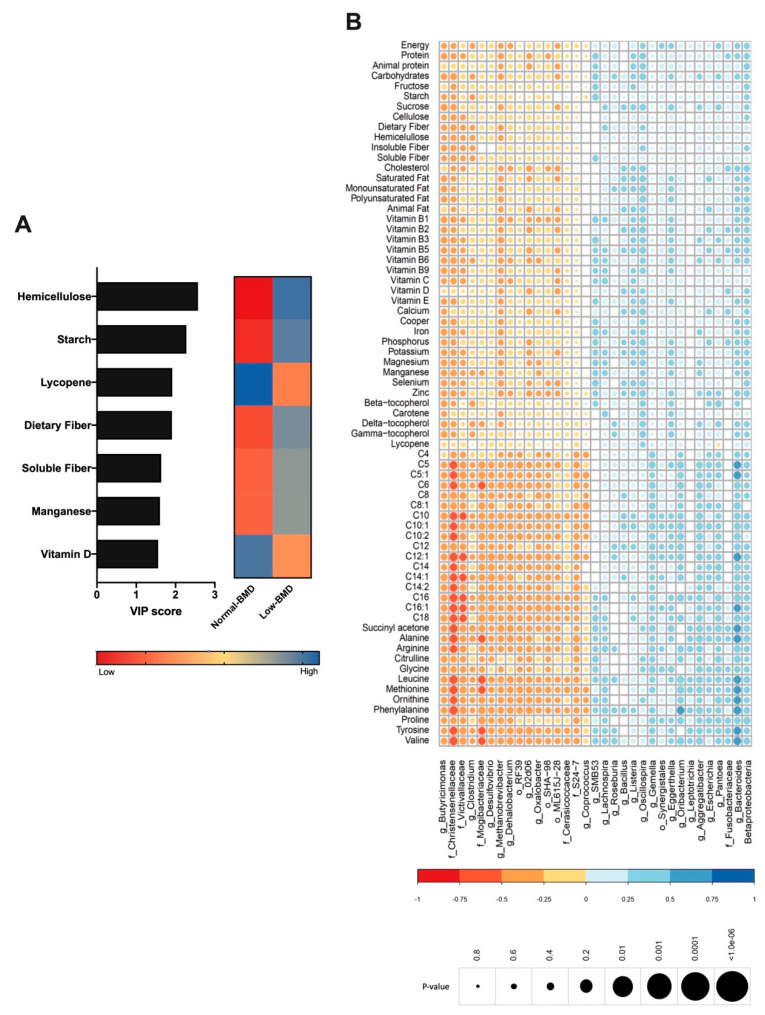
Contribution of dietary components to the composition of gut microbiota. (**A**) VIP analysis represents the relative contribution of nutrient consumption to the variance between normal-BMD and low-BMD. The high value of the VIP score indicates the great contribution of the nutrient consumption to the group separation. The blue and red boxes on the right indicate whether the consumption is increased (blue) or decreased (red). The low-BMD group diet was characterized by higher fiber intake, starch, and manganese consumption. The normal-BMD group was characterized by lycopene and vitamin D intake. (**B**) Correlation heatmap. Blue and red color indicates an increase and decreased correlation, respectively. The size of each dot was associated with the *p*-value, where a big circle represents a smaller *p*-value. *Lachnospiraceae* abundance was positively correlated with fiber consumption and in a negative manner with vitamin D intake. Calcium consumption was negatively correlated with *Butyricimonas*. Lycopene consumption was decreased in the low-BMD group, and positively correlated with *Oscillospira* and negatively correlated with *Pantoea* genus abundance.

Diet is an important driver of gut microbiota; therefore, the dietary intake was analyzed in both groups and correlated with microbiota composition. The low-BMD group´s diet was characterized by higher fiber intake (hemicellulose, dietary fiber, and soluble fiber), starch, and manganese consumption (Figure 4A). In contrast, the components in the normal-BMD group were characterized by lycopene and vitamin D intake (Figure 4A). *Lachnospiraceae*, *Vagococcus*, and *Dialister* positively correlated with fiber consumption. Vitamin D intake was negatively correlated with *Lachnospiraceae* and positively correlated with *Oscillospira*. Calcium consumption was negatively correlated with *Butyricimonas* (genus). Lycopene consumption was decreased in the low-BMD group, and positively correlated with *Oscillospira* and negatively correlated with *Pantoea* genus abundance (Figure 4B).

### 3.5. Association Between Serum Metabolites with Bone Mineral Density, Intestinal Microbiota, and Vitamin D Deficiency 

**Figure 5:**
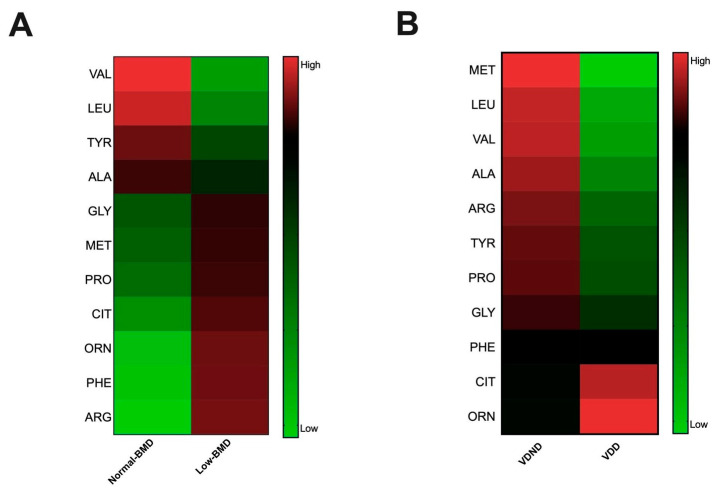
Association between serum metabolites with bone mineral density and vitamin D deficiency. (**A**) Amino acid concentration heatmap in each group; red and green indicate an increased and decreased concentration, respectively; the low-BMD group had a lower valine and leucine concentration than the normal-BMD group. (**B**) Amino acid concentration heatmap in each group, red and green indicate increase and decreased concentration, respectively. Postmenopausal no vitamin D-deficient women (VDND) had higher serum concentrations of valine and leucine, tyrosine, alanine, and proline than postmenopausal vitamin D-deficient women (VDD).

As mentioned, several studies further linked gut microbiota to serum amino acids, which have been implicated in metabolic diseases; hence, the serum metabolites were determined in both groups and correlated with microbiota composition. BCAAs, leucine and valine, and aromatic amino acids (AAAs), tyrosine, were negatively correlated with the *Christensenellaceae* and *Mogibacteriaceae* family and positively correlated with *Bacteroides* (Figure 4B). Interestingly, the low-BMD group had lower BCAAs compared to the normal-BMD group (Figure 5A).

Vitamin D deficiency is related with bone disorders, including low BMD, and increases fracture risk. Remarkably, postmenopausal women deficient in vitamin D have higher concentrations of citrulline and ornithine than deficient women with higher serum concentrations of BCAAs, glucogenic, and AAAs (Figure 5B).

### 3.6. Association Between Vitamin D Deficiency and Bacterial Community Composition

**Figure 6:**
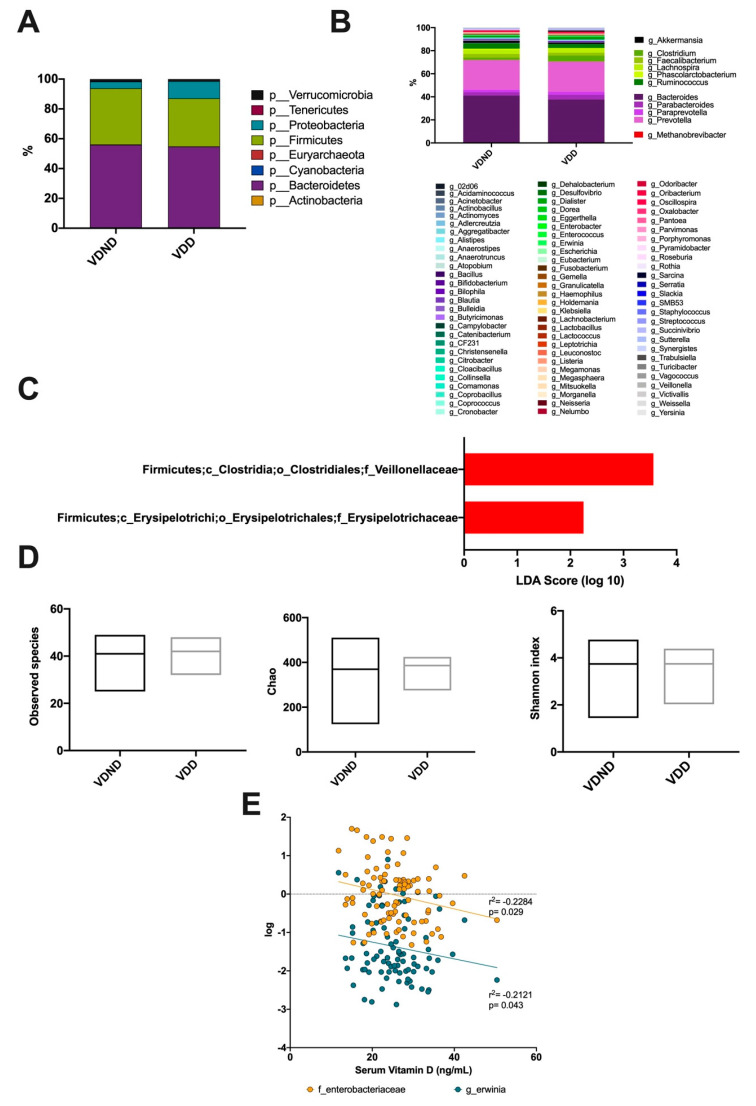
Association between vitamin D deficiency and bacterial community composition. (**A**) A total of seven phyla dominated the microbiota composition in postmenopausal no vitamin D-deficient women (VND) and postmenopausal vitamin D-deficient women (VDD); the predominant phyla were *Bacteroidetes*, colored purple, followed by *Firmicutes*, colored green. (**B**) At the genus level, the most abundant taxa were *Akkermansia*, *Clostridium*, *Faecalibacterium*, *Lachnospira*, *Phascolarctobacterium*, *Ruminococcus*, *Bacteroides*, *Parabacteroides*, *Paraprevotella*, *Prevotella*, and *Methanobrevibacter*. (**C**) Bacterial taxa differentially represented between groups identified by linear discriminant analysis (LDA) effect size. Only taxa with an alpha value of 0.05 and with an LDA score of at least 2 are shown. The intestinal microbiota of women in the vitamin D-deficient group (VDD) had significantly higher proportions of *Erysipelotrichaceae* and *Veillonellaceae* compared to the vitamin D non-deficient group (VDND). (**D**) No significant differences were found between the groups in observed species, richness (Chao), or diversity (Shannon index). (**E**) Vitamin D levels negatively correlates with *Enterobacteriaceae* (r = −0.262; 95% CI (−0.444-0.060), *p* = 0.011) and *Erwinia* (r = −0.212; 95% CI (-0.406−0.0008), *p* = 0.043).

Regarding gut microbiota, eight phyla were detected in both groups. The prevalent phyla were *Firmicutes* and *Bacteroidetes*, succeeded by *Proteobacteria*, *Verrucomicrobia*, *Tenericutes*, *Cyanobacteria*, *Actinobacteria*, and *Euryarchaeota*, which were limited (Figure 6A). A total of 11 genera were detected in both groups: *Akkermansia*, *Clostridium*, *Faecalibacterium*, *Lachnospira*, *Phascolarctobacterium*, *Ruminococcus*, *Bacteroides*, *Parabacteroides*, *Paraprevotella*, *Prevotella*, and *Methanobrevibacter* were the most abundant at almost 87% (Figure 6B). The intestinal microbiota of women in the group with VDD had significantly higher proportions of *Erysipelotrichaceae* and *Veillonellaceae* compared to VDND (Figure 6C). No significant difference was observed in diversity indices between the two groups (Figure 6D). Interestingly, serum vitamin D levels negatively correlated with *Enterobacteriaceae* family abundance (r2 = −0.2284; *p* = 0.029) and *Erwinia* genus abundance (r2 = −0.2121; *p* = 0.043) (Figure 6E).

## 4. Discussion

The gut microbiota is recognized as a significant contributor to many human chronic diseases [26]. Otherwise, changes in the gut microbiota have been associated with bone homeostasis and the quality of the bone tissue [27]. However, the specific relationship among the composition of gut microbiota, dietary nutrients, and serum metabolites that could impact bone health remains a subject that has been scarcely known. A pilot multi-omic profiling was performed to explore the relationship among diet, the serum metabolome, and gut microbiota, comparing them between normal- and low-BMD postmenopausal women groups. 

Our results are consistent with other studies suggesting that low-BMD subjects have fewer observed species than the normal-BMD group [28,29]. The mechanisms underlying the decrease in observed species in low-BMD women remain elusive. It suggested that aging, dental deterioration, salivary function, digestion, and intestinal transit time impacts diet diversity, affecting the number of species (diversity) in the microbiota [30]. Another possible explanation is that older people become less active; hence, their metabolism slows down, and their energy requirements decrease, which means they eat less [24]. In our study, the low-BMD group included mainly older women compared to the normal-BMD group, contributing to lower observed species. 

In the present study, the abundance of *Klebsiella* and *Erwinia* genus belonging to γ-*Proteobacteria* phylum were increased in the group with low-BMD compared with the normal group. This finding coincides with that recently reported by He et al. in postmenopausal women with osteopenia [29]. Interestingly, during intestinal dysbiosis, it has been observed that mice have less femur bending strength, deterioration in the properties of bone tissue, and a greater abundance of *Proteobacteria* [31]. He et al. suggest that the abundance of this phyla is negatively associated with bone mineral density; this could make sense because some members of *Proteobacteria* phyla have been associated with dysbiotic processes that lead to inflammation, which correlates with osteoclast activation, thus affecting bone health [13]. 

Wang et al. reported the relationship between osteoporosis/osteopenia and the intestinal microbiota. The authors point out that composition and bacterial diversity is different in patients with OP and osteopenia than in healthy subjects [24]. Li et al. reported that low-BMD individuals had a low number of OTUs (operational taxonomic units), the abundance of *Lachnospiraceae* in people with low-BMD, and a positive correlation between BMD and T-score [28]. Recently, Das et al. found an abundance of *Clostridium cluster* XlVa, *Lactobacillus*, *Actinomyces*, and *Eggerthella* in OP patients compared to healthy donors [32], which is contradictory with our findings. However, differences in several factors, such as sample size, ethnicity, diet, age, and sex of participants, may be the reason for these inconsistencies. The present study included only women between 45 and 80 years old; the other studies also evaluated men, and participants’ age was over 60 years. On the other hand, the women in this study did not have chronic non-communicable diseases. 

Otherwise, *Actinobacteria* was significantly more abundant in the normal-BMD group. Interestingly, some members of the *Actinobacteria* phylum have been positively associated with bone density. The possible mechanism would be related to the decrease in intestinal inflammation and the improvement of the intestinal barrier [33]. Additional studies will be necessary to understand these mechanisms. 

The gut microbiota composition changes related to age include a decrease in saccharolytic bacteria like *Bifidobacterium*, the proportion of *Bacteroides*/*Prevotella*, *Lactobacilli*, and *Faecalibacterium prausnitzii*. Age also increases the abundance of *Ruminococcus*, *Atopobium*, and certain *Proteobacteria*, which contains several pathobionts [30,34]. Aging progression takes many sides of the human body’s biological variation, which leads to functional decline and an increased incidence of infection in the gut of older people [30]. However, the characteristics of the older person’s gut microbiota seem to be promoted by chronic low-grade inflammation caused by an imbalance between pro- and anti-inflammatory pathways, a typical feature of the aging process called “inflammageing”. Recently, Cicero et al. demonstrated in elderly subjects who consumed a symbiotic formula containing *Lactobacillus plantarum* PBS067, *Lactobacillus acidophilus* PBS066, and *Lactobacillus reuteri* PBS072 decreased serum markers associated with inflammation, such as C-reactive protein and tumor necrosis alpha-factor, as well as several risk factors associated with metabolic syndrome [35]. Interestingly, in the present study, only the group of middle-aged women (age 45–59 years) with low-BMD presented changes markedly associated with aging.

Diet is involved in modifying bone health and is an important driver in the intestinal microbiota composition. Hence, nutrients influence gut microbiota composition, and, in turn, microbiota produces metabolites via the degradation of nutrients that may affect several cellular functions and modulate the immune response [30]. Some of these metabolites have been implicated in type 2 diabetes and cardiovascular diseases [8]; for example, *Prevotella copri* and *Bacteroides vulgatus* have been associated with increased concentrations of BCAAs and with the presence of IR [7]. Otherwise, the association between BCAAs and muscle and fat mass may be related to the potential effects on bone health [7], although both are critical for the maintenance of bone strength and density [36]. In the case of AAs, the evidence suggests a favorable role in bone health, mainly by promoting osteoblast proliferation and differentiation and acting as signaling molecules in bone cells [37]. However, their associations with BMD and microbiota composition have not been established in postmenopausal women. In the present study, postmenopausal women without vitamin D deficiency and women with high bone mineral density showed higher concentrations of BCAAs—leucine and valine—and AAA—tyrosine—levels. According to previous metabolic studies, our results agreed that some circulating AAs revealed potential associations with BMD in women [38,39,40,41,42]. Remarkably, these amino acids correlated positively with the abundance of *Bacteroides*. Recently, Ponsuksili et al. correlated *Bacteroides* abundance with several miRNAs and transcripts involved with bone metabolism and inflammatory response [43]. *Bacteroides fragilis* evoke an anti-inflammatory response by inducing Treg cells. Therefore, microbiota composition may impact bone health by affecting T cell differentiation, eliciting an anti-inflammatory response by inducing Treg cells. Thus, it is possible that microbiota composition can impact bone health by influencing T cell differentiation [44].

As mentioned above, diet is an important driver for the intestinal microbiota composition. In our study, lycopene consumption was decreased in the low-BMD group, and positively correlated with *Oscillospira* and negatively correlated with *Pantoea* genus abundance. Carotenoids are natural pigments that are considered key players in the regulation of bone metabolism [45]. Carotenoids are classified, based on their functions as xanthophylls (including lutein and zeaxanthin), and carotenes (such as α-carotene, β-carotene, and lycopene). The latter is one of the main carotenoids and is among the best-documented nutraceutical compounds with a significant health benefit. Our observations are in line with other previous findings demonstrating that a diet rich in carotenoids has a positive impact over the BMD [45] and that lycopene influences osteoblast differentiation [46,47]. Furthermore, *Oscillospira* abundance is associated with microbial diversity, leanness, and high levels of HDL [48]; meanwhile, *Pantoea* is a potentially pathogenic bacteria belonging to γ-*Proteobacteria* phylum and *Enterobacteriaceae* family associated with inflammatory responses [49]. The results suggest that lycopene dietary intake and supplementation benefit Mexican postmenopausal women, increasing BMD and lowering inflammatory responses. However, clinical studies are necessary to verify lycopene’s therapeutic effects on the BMD.

Finally, bone tissue mineralization and intestinal microbiota are determined by several factors, including vitamin D levels [50]. Previously reported studies on the Mexican population described a high prevalence of vitamin D deficiency in postmenopausal women [15]. Here, for the first time, vitamin D-deficiency-associated alterations in the microbiota in postmenopausal women were reported. Although several uncontrolled or human observational studies have been published, those studies are on experimental animals [51,52]. Interestingly, the intestinal microbiota of women with vitamin D-deficiency was related to *Erysipelotrichaceae* and *Veillonellaceae* abundance compared to the vitamin D non-deficient group. Interestingly, both taxa are affected in patients with cystic fibrosis-associated vitamin D deficiency [53]. The *Erysipelotrichaceae* family possess opportunistic pathogenic bacteria and have been correlated to obesity, colorectal cancer, and inflammation. Collectively, we speculate that the observed increase in *Erysipelotrichaceae* and *Veillonellaceae* in vitamin D deficiency in postmenopausal women has the potential to contribute to an increased risk of inflammation and metabolic disorders. This speculation is worth further investigation with long-term follow-up of postmenopausal women with vitamin D deficiency. 

Our study has some limitations but several key strengths. Even though we analyzed a modest sample size, this is the first study of diet, the microbiota, and the metabolome conducted in postmenopausal women with a low BMD. The information on the diet was collected from a self-reported questionnaire including 116 items. So, the error derived from the questionnaire may minimize other possible correlations in our measurements. Additionally, all the participants were recruited from the central region of Mexico and probably have similar dietary habits. The gut microbiota residing in the intestine is a complex community. Hence, the dynamics of the composition and function are influenced by non-modifiable factors, such as age, sex, and geographical location, and modifiable factors, such as diet, illness, and medications, especially antibiotics. Only postmenopausal women were selected, excluding women with exposure to antibiotics or hormone replacement therapy within six months before the collection of the fecal sample and excluding women who had non-communicable diseases. A limitation of our study is that the sampling was cross-sectional (third HWCS assessment). Longitudinal studies are required to determine whether changes in dietary patterns affect the metabolism mediated by the gut microbiota and whether these variations can influence bone health.

## 5. Conclusions

By performing a multi-omic analysis in a subsample of a widely phenotyped cohort study, for the first time, microbiome-mediated relationships between circulating metabolites and diet with low-BMD were identified. The findings of our analysis provide helpful evidence to elucidate the underlying microbiota–diet–metabolites relevant mechanism in loss of bone mineral density.

## Figures and Tables

**Table 1:** Characteristics of selected postmenopausal women of the Health Workers Cohort Study.

	Total	Normal-BMD	Low-BMD	*p*-value
	*n* = 92	*n* = 34	*n* = 58	
Age(years) *	63.0(58.0–70.0)	60.5(56.0–65)	68.0(60.0–74.0)	0.0010
Age Categories, %				
45–59 years	31.5	47.1	22.4	0.0138
60–74 years	57.6	52.9	60.3	0.4882
>74 years	10.9	-	17.2	0.0104
BMI (kg/m^2^) *	25.8(22.7–27.8)	26.2(23.8–29.6)	25.2(22.5–27.6)	0.1039
Nutritional Status, %				
Overweight	41.3	41.2	41.4	0.9850
Obesity	13.0	20.6	8.6	0.0989
Waist circumference (cm) *	87.0(81.0–94.5)	89.0(82.0–94.0)	86.0(81.0–95.0)	0.2453
Body fat proportion *	42.6(38.9–46.8)	42.7(39.1–37.3)	42.4(38.8–45.7)	0.7116
Leisure time physical activity (hours/week) *	1.5(0.4-3.5)	1.2(0.4–3.5)	1.5(0.4–4.5)	0.6783
Active (≥2.5 h/week), %	38.0	32.4	41.4	0.3908
Smoking, %				
Current	4.4	2.9	5.2	0.6157
Past	27.2	29.4	25.9	0.6613
Glucose (mg/dL) *	97.0(89.0–102.5)	97.0(89.0–104.0)	97.0(90.0–102.0)	0.6561
Impaired Glucose tolerance (≥100–125 mg/dL), %	17.4	8.8	23.6	0.0965
Type 2 diabetes, %	43.5	55.9	34.6	0.0658
Total cholesterol (mg/dL) *	94.5(66.5–154.2)	113.0(78.0–179.0)	88.0(56.0–145.0)	0.0327
Triglyceride (mg/dL) *	137.0(110.5–193.5)	139.0(114.0–238.0)	136.0(108.0–185.0)	0.5280
HDL-C(mg/dL) *	53.7(46.0–65.9)	49.4(42.4–61.9)	55.3(48.0–68.8)	0.0856
LDL-C(mg/dL) *	119.7(89.9–137.6)	119(88–135)	122(91–139)	0.4641
Systolic blood pressure (mmHg) *	117(106–131)	117(106–130)	118(106–134)	0.9098
Diastolic blood pressure (mmHg) *	74.0(66.5–79.0)	74(69–80)	74(66–78)	0.1737
Femoral neck- BMD (g/cm^2^) *	0.78(0.70–0.87)	0.88(0.86–0.93)	0.74(0.63–0.78)	<0.001
Lumbar spine- BMD (g/cm^2^) *	0.95(0.84–1.07)	1.02(0.95–1.13)	0.89(0.81–1.00)	0.0002
Serum 25(OH)D levels (ng/mL) *	25.6(20.4–28.8)	25.5(22.4–30.2)	25.6(20.2–28.5)	0.9825
Deficiency Vitamin D, %	20.9	18	22.8	0.5860
Diet				
Total energy intake (kcal/d) *	1420(1164–1858)	1464(1185–1869)	1409(1141–1730)	0.5123
Carbohydrates (% of energy total) *	65.2(60.5–70.0)	63.9(59.9–67.9)	66.8(60.8–70.7)	0.0864
Protein (% of energy total) *	12.2(10.8–14.5)	12.4(11.2–14.9)	12.1(10.3–14.2)	0.3158
Alcohol intake (g/1000 kcal intake) *	0.8(0.0–2.5)	0.7(0.04–1.6)	0.8(0.0–3.0)	0.9052
Vitamin D intake (IU/1000 kcal intake) *	72.6(37.1–119.4)	76.0(44.7–142.2)	72.0(31.4–110.6)	0.2160
Calcium intake (mg/1000 kcal intake) *	414.5(33.8.5–548.3)	472.1(339.1–586.6)	397.2(337.9–514.2)	0.1590
Lycopene (µg/1000 kcal intake) *	2507(1326–4100)	2739(1256–3707)	2262(1346–4267)	0.8970
Hemicelullose (g/1000 kcal intake) *	3.1(2.1–5.1)	2.5(1.9–4.2)	3.5(2.2–5.3)	0.0480
Starch (g/1000 kcal intake) *	46.6(27.2–58.2)	46.8(28.0–59.5)	48.2(26.6–57.1)	0.8460
Soluble dietary fiber (g/1000 kcal intake) *	4.4(3.6–5.5)	4.2(3.6–5.3)	4.6(3.4–5.6)	0.4090
Manganese (mg/1000 kcal intake) *	1.5(1.3–2.0)	1.4(1.2–1.9)	1.7(1.4–2.0)	0.1020
Caffeine (g/day)*	51.2(6.6–116.5)	50.6(13.1–97.7)	55.2(5.7–116.8)	0.6535
Dietary fiber (g/1000 kcal intake) *	16.7(13.8–21.8)	15.2(12.7–18.3)	17.8(14.9–22.5)	0.0216
Magnesium (mg/1000 kcal intake) *	206.5(180.8–224.6)	198.6(172.1–222.8)	209.5(185.9–227.2)	0.1793
Phosphorus (mg/1000 kcal intake) *	632.2(551.1–741.6)	665.3(572.7–788.7)	603.1(539.3–699.7)	0.1476
Potassium (mg/1000 kcal intake) *	1884(1628–2276)	1856(1620–2098)	1926(1634–2335)	0.2930
Zinc (mg/1000 kcal intake) *	3.6(3.1–4.2)	0.7(0.04–1.6)	0.8(0.0–3.0)	0.6506

* Continuous variables are presented as Median (25th–75th percentiles).
